# Long-Lived Plasma Cells Secrete High-Affinity Antibodies Responding to a T-Dependent Immunization in a Teleost Fish

**DOI:** 10.3389/fimmu.2019.02324

**Published:** 2019-10-02

**Authors:** Liting Wu, Shengli Fu, Xiaoxue Yin, Zheng Guo, Anli Wang, Jianmin Ye

**Affiliations:** Guangdong Provincial Key Laboratory for Healthy and Safe Aquaculture, Institute of Modern Aquaculture Science and Engineering, School of Life Sciences, South China Normal University, Guangzhou, China

**Keywords:** Ictalurus punctatus, long-lived plasma cells, affinity, anterior kidney, affinity-driven selection

## Abstract

The recent discovery of long-lived plasma cells (LLPCs) in mammals, which provide a constant expression of specific high-affinity antibodies that mediate humoral memory, has caused a dramatic paradigm shift in the study of immunity and vaccine development. In teleost fish, there are few studies regarding the association between LLPCs and antibody production, and the affinity of the antibodies secreted by the LLPCs is poorly understood. In the present study, channel catfish (*Ictalurus punctatus*) were immunized with trinitrophenylated-keyhole limpet hemocyanin (TNP-KLH) to examine TNP-specific antibody titers, affinities, antibody-secreting cell (ASC) dynamic changes, and especially the affinity of secreted antibodies by LLPCs post-immunization. We demonstrated that TNP-specific LLPCs were generated starting at week 4 post-immunization, achieved a maximal number at week 8, and maintained a comparable level throughout the 18-week post-immunization period, which was correlated with the dynamics of serum antibody titers and affinity maturation in the response. The LLPCs appeared to mostly reside within, or migrate to, the anterior kidney (bone marrow-like tissue in mammals), but a small portion was also located in the spleen and peripheral blood. The antibodies produced by the LLPCs possessed high affinities, indicating that the generation and development of LLPCs were driven by affinity selection in teleosts. Collectively, the results of this study provide insights toward the evolutionary understanding of the affinity-dependent mechanism of LLPC generation and development.

## Introduction

In mammals, the existence of long-lived plasma cells (LLPCs) demonstrates a new mechanism to maintain humoral immunity. LLPCs are considered to be a new kind of “memory-providing cell” and survive independent of antigen (1–4). LLPCs differ from short-lived plasma cells (SLPCs) in that LLPCs primarily arise weeks after antigen exposure, with accumulated V region mutations due to affinity-selection within the germinal center (1, 5–7). These LLPCs ultimately reside in the bone marrow for extended periods and account for over 80% of the antibodies present in serum (8). Serum antibodies are initially produced by antibody-secreting cells (ASCs) in the draining lymph nodes and spleen, which transiently peak and then decline within a few weeks of antigenic stimulation following a single immunization (5, 8). The phenomenon of affinity maturation not only reveals that antibody production persisted for months and even years after immunization but also indicates that long-lived high-affinity antibodies reside in the bone marrow, which demonstrates that LLPCs secrete antibodies to sustain the serum titer and high affinity in the late immune stage (9–13).

In teleost fish, ASCs had been presumed as a uniform population of plasma cells. Recently, comparable to that of mammals, B cell diversity was described in teleosts, harboring plasmablasts and plasma cells (14–18). According to cell longevity, the presence of both long-lived and SLPCs was demonstrated in trout (17). Most importantly, these B cell subpopulations were not uniformly distributed throughout all immune tissues, with LLPCs, thus far, being observed exclusively within the anterior kidney, and plasmablasts being found, to varying degrees, in all immune tissues examined (17, 19). The anterior kidney is a key organ for immunity and the major site of hematopoiesis, which resembles the bone marrow in mammals (20–23). Late in the response, these LLPCs were demonstrated to only reside in the anterior kidney, which was able to continuously secrete specific antibodies after 15 days in *in vitro* culture (17). As in mammals, these LLPCs provided a similar constant expression of specific antibodies over a 35-week period post-immunization in trout (19) and appeared to be primarily or entirely responsible for the long-term antibody responses in teleost (24). Although the form of the response may not be the same as in mammals (incorporating isotype switching, logarithmic increase in titer, and affinity), affinity maturation was still observed to occur in teleost over a TD antigen response (25–28). When affinity maturation occurred, the high-affinity antibodies appeared later, while the early appearance of low-affinity antibodies decreased over the response (16, 17, 19). Thus, there was a correlation between a significant increase in antibody affinity over the response and the late appearance of LLPCs within the anterior kidney (29). This seems to indicate that teleost LLPCs may secrete high-affinity antibodies in teleost fish.

LLPCs are crucial to providing lifetime protection against pathogenic infections in teleost fish, similar in mammals, which is important for vaccine development for aquaculture. Until now, studies in channel catfish exposed to the protozoan parasite, *Ichthyophthirius multifiliis*, have suggested that long-term protective immunity seems to be provided by IgM^+^ memory B cells rather than long-lived, non-dividing plasma cells (18, 30, 31). Our previous reports have demonstrated that, as in rainbow trout, affinity maturation occurs in catfish and high-affinity antibody subpopulations appear post-immunization with a T cell-dependent (TD) antigen, trinitrophenylated-keyhole limpet hemocyanin (TNP-KLH) (28). Thus, we proposed that LLPCs may exist in channel catfish, similar to rainbow trout. However, it is poorly understood if teleost LLPCs secrete high-affinity antibodies to sustain the high-affinity antibody levels in the immune response as in mammals. In this paper, we aimed to examine the existence of LLPCs and explore the affinity of specific antibodies secreted by LLPCs in channel catfish (*Ictalurus punctatus*). With TNP-KLH immunization, the TNP-specific titers, affinities, ASCs dynamic changes, and especially the affinity of secreted antibodies by LLPCs post-immunization were studied. Data obtained here showed that anti-TNP-specific LLPCs migrated and survived in the anterior kidney throughout a period of 18 weeks, which correlated with the dynamics of affinity maturation of the serum antibody response. The phenomenon of LLPCs of teleosts secreting high-affinity antibodies provided evolutionary evidence that affinity-driven selection plays a significant role in the generation and development of LLPCs.

## Materials and Methods

### Fish

Channel catfish (*I. punctatus*) were obtained from Guangdong Catfish Breeding Farm in Guangzhou (Guangdong, China), weighing 600 ± 150 g. They were maintained in a semi-automatic circulating water system (28) at 28 ± 2°C. Fish were fed for 3 weeks prior to any experiment, and all animal protocols were reviewed and approved by the University Animal Care and Use Committee of the South China Normal University.

### Antigens and Immunization

The immunogen (TNP-KLH) was prepared as described previously (28, 32). TNP_1_-BSA and TNP_8_-BSA (19, 27) were employed as the antigen for antibody titer determinations and affinity and ELISPOT analysis.

Channel catfish were anesthetized with a 0.04% solution of 3-aminobenzoic acid ethyl ester (MS-222; Aladdin, China) prior to immunization. Sixty fish were immunized with 100 μg of TNP-KLH per fish by intraperitoneal injection in a final volume of 100 μL [TNP-KLH in PBS emulsified 1:1 with Freund's complete adjuvant (FCA; Sigma-Aldrich, St. Louis, MO, USA)]. The control group was injected with PBS emulsified with FCA. In order to prepare the serum at 0, 2, 4, 6, 8, 10, 14, and 18 weeks post-immunization (p.i.), five fish were selected at random from each group. Fish were anesthetized with MS-222 (Aladdin, China), and 1 mL of blood was collected by venipuncture from the caudal vessel and then placed in a sterile 1.5 mL tube. The specific individuals were tracked over the entire immunization period. The blood was centrifuged at 4°C, 500 × g for 10 min, and the serum was collected and stored at −80°C.

### Isolation of Leukocytes From Peripheral Blood, Spleen, and Anterior Kidney

Leukocyte isolation was performed as previously described (17) with some modifications. Briefly, channel catfish were anesthetized in water with ~0.04% MS-222 at 0, 2, 4, 6, 8, 10, 14, and 18 weeks post-immunization. Peripheral blood (PBL) was immediately collected from the caudal vein with a 2.5 mL syringe. After centrifugation at 4°C, 500 × g for 10 min, the serum was discarded. The remaining blood cells were diluted 6-fold of the original volume of blood with RPMI-1640 (Gibco, USA) at 25°C, and the suspended cells were then gently put on ice. Anterior kidney (AK) and spleen (SPL) were dissected from the fish and placed in a sterile plastic culture dish containing 5 mL of RPMI-1640 with 100 U/mL of penicillin G and 100 mg/mL of streptomycin (Sigma-Aldrich, USA), respectively. To obtain single-cell suspensions, tissues were teased apart with sterile dissecting scissors, then transferred to sterile bottles and repeatedly aspirated using a 2.5 mL syringe until no big tissue remained. The single-cell suspension was transferred to a sterility tube, and RPMI-1640 (~10 mL) was added to the total volume. Each tissue suspension was slowly layered on an equal volume of Histopaque 1077 (Sigma-Aldrich, USA) in 50-mL conical centrifuge tubes and centrifuged at 500 × g for 40 min at 4°C. Leukocytes collected from the interface layer were washed three times by centrifugation in RPMI-1640. Viability was determined by trypan blue (Sigma-Aldrich, USA), and the cells were resuspended to a concentration of 1 × 10^7^ cells/mL in tissue culture medium (TCM). The TCM (33) consisted of basic RPMI-1640 with 1% L-glutamine (Sigma-Aldrich, USA), 11 μg/mL sodium pyruvate (Sigma-Aldrich, USA), 10% fetal bovine serum (Gibco, USA), 50 μg/mL gentamycin (Sigma-Aldrich, USA), 5 μM 2-mercaptoethanol (Sigma-Aldrich, USA), 1× non-essential amino acids (Sigma-Aldrich, USA), and 10 μg/mL each of adenosine, cytosine, uracil, and guanine (Sigma-Aldrich, USA).

### *In vitro* Cell Culture With Hydroxyurea (HU)

The leukocytes were cultured *in vitro* at a density of 1 × 10^6^ cells in 100 μL of TCM per well in 96-well plates (Thermo Fisher, Waltham, MA, USA), which were put into the incubator culture chamber M-624 (C.B.S Scientific, San Diego, CA, USA) for 7 or 15 days in an atmosphere of 10% O_2_, 10% CO_2_, and 80% N_2_ (19). In order to distinguish plasma cells from plasmablasts, hydroxyurea (HU) was used at 100 mM as previously described (17). HU is an inhibitor of DNA replication, which functionally distinguishes between HU-sensitive plasmablasts and HU-insensitive plasma cells.

### Antibody Titer and Affinity Determinations

A standard antibody titration ELISA was used to detect the anti-TNP sera titers of channel catfish as described in trout (27). The microplate reader Multiskan FC (Thermo Fisher, USA) was used to measure the optical density (O.D.) rate at 405 nm, since the substrate was 2,2′-Azinobis-(3-ethylbenzthiazoline-6-sulphonate) (ABTS). A unit of antibody activity was defined as the volume of sample required to produce an O.D. rate of half the maximum rate (34). Anti-TNP sera antibody affinities were assessed by the ratio of TNP_1_-BSA to TNP_8_-BSA O.D. values, with high valence TNP_8_-BSA combined with more low affinity antibodies. This easier and more sensitive detection was remolded as described previously (35) with some modifications. In contrast to the previous method, which used the binding ratio of different valency antigens to represent antibody maturity by flow cytometric analysis, the different valency antigen ratios were employed (TNP_1_-BSA/TNP_8_-BSA, and the method was shortened for T1/T8), named T1/T8 affinity ELISA. The antibody titers of cell supernatants collected from the leukocytes cultured *in vitro* were much lower than those of serum antibodies; however, their affinities were able to be detected by the T1/T8 affinity ELISA. Different from the standard antibody titration ELISA, the substrate used in T1/T8 affinity ELISA was tetramethylene benzidine dihydrochloride (TMB; Sigma-Aldrich, USA), according to the previously described protocol (36). In the analysis of sera antibody affinities, an affinity partitioning ELISA was employed to detect the differential dynamics of antibody subpopulations over the course of the response at weeks 2, 6, and 18 post-immunization (25, 27).

### ELISPOT to Dissect ASC Responses

ASCs were enumerated by ELISPOT analysis as previously described (17). Briefly, after activation with methanol, the polyvinylidene fluoride (PVDF) membrane was washed three times with PBS. PBS containing 50 μg/mL TNP_8_-BSA was incubated with the membrane for 2 h on a shaker at 25°C. The membrane was washed with PBS for three times and then blocked with the blocking solution (0.5% BSA in PBS) for 2 h at 25°C. The membrane was washed three times, and the membrane wrapped with parafilm was then inserted into a dot-blot apparatus (BioRad, USA), and 10^4^-10^6^ cells, *ex vivo* or cultured *in vitro* for 15 days (17) (the cell supernatants of these cells were collected first before adding into dot-blot apparatus), per well were added in a total volume of 200 μL and then cultured at 25°C overnight for 15 h. The membrane was subsequently removed, wiped with Kimwipes (Kimtech Science Kimwipes, USA), washed with PBS three times, and incubated with gentle shaking in a blocking solution for 30 min. The membrane was then washed with PBS and incubated with gentle shaking at 25°C for 2 h in PBS containing 0.5 μg/mL mouse-anti-channel catfish IgM heavy chain (MAF13) (18, 30). The membrane was then washed with PBS three times and incubated with gentle shaking at 25°C for 2 h in PBS containing 0.125 μg/mL goat-anti-mouse IgG conjugated with HRP (SouthernBiotech, Tuscaloosa, AL, USA). The membrane was washed three times and then incubated with 3-amino-9-ethylcarbazole (AEC; 8 mg) (Sigma-Aldrich, USA) in 0.05 M acetate buffer with 0.04% H_2_O_2_. AEC at a concentration of 40 mg/mL was first solubilized in dimethylformamide before dilution in the acetate buffer. The reaction was terminated by flushing with tap water, and then the membrane was air dried. The ASCs were enumerated using a stereoscope SteREO Discovery.V8 (Zeiss, Germany), and spots were counted on the enlarged photographs. One spot represented an anti-TNP specific ASC. The size of the ELISPOTs was analyzed by Image J software (National Institutes of Health, USA). The bigger the spot size, the stronger was the secreting ability of the cell.

## Results

### TNP-Specific Antibody Response to TNP-KLH

**Figure 1:**
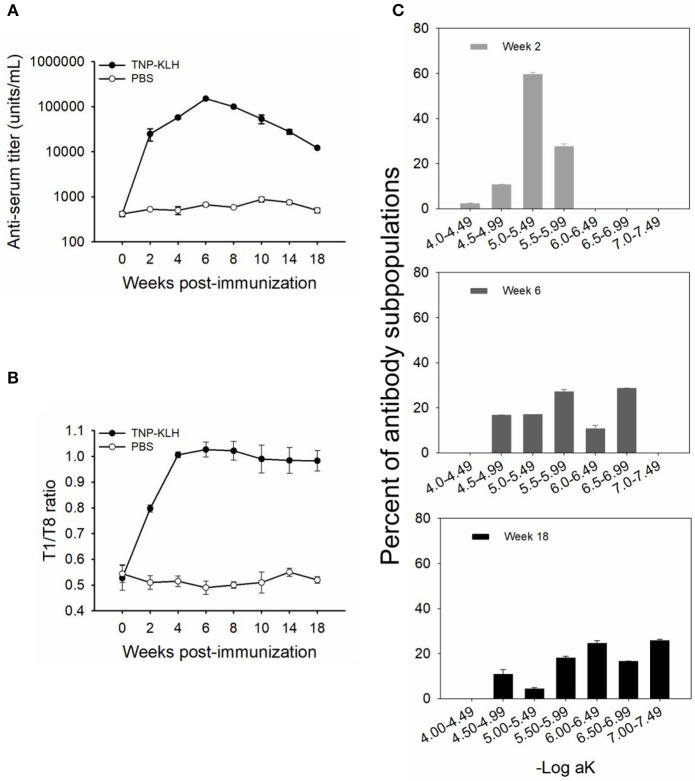
Anti-TNP antibody titers and affinities induced by trinitrophenylated-keyhole limpet hemocyanin (TNP-KLH) (*n* = 5). The dynamics of anti-TNP antibody titers were detected by standard antibody titration ELISA **(A)**. The T1/T8 ratio was used to present the dynamic of antibody maturation **(B)**, and affinity partitioning ELISA **(C)** was used to detect the differential dynamics of antibody subpopulations at weeks 2, 6, and 18 post-immunization. Week 0 was bled prior to immunization, and PBS was immunized as the control. Data represent the mean ± standard deviation (SD) of five individual fish at each time point and are representative of three independent experiments.

A group of five catfish was immunized with TNP-KLH, and sera titers and affinities to TNP were monitored at weeks 0, 2, 4, 6, 8, 10, 14, and 18 post-immunization. It can be seen that TNP-specific titers increased rapidly at week 2 (24,869 units/mL) and reached the peak at week 6 (150,715 units/mL) with logarithmic increases and then began to slowly decline and maintained a stable level during the late immunization (Figure 1A). The control fish (immunized with PBS) did not exhibit any titers significantly greater than those seen at week 0.

The affinity of TNP-specific antibodies detected by the T1/T8 ratio ELISA showed that the affinity increased at every time point through week 10, 4 weeks past the point at which the maximal titers were observed (Figure 1B), indicating that affinity maturation occurred in channel catfish. Furthermore, at the late response, the antibody affinity was maintained at a high level, with a ratio of ~0.98 at week 18, while there were no changes in control fish (immunized with PBS) (Figure 1B). In addition to the average affinity revealing affinity maturation, the examination of the behavior of the individual affinity subpopulations was considerably more informative (Figure 1C). The average percent (% of whole titer) of individual affinity subpopulations at weeks 2, 6, and 18 was monitored by an affinity partitioning ELISA (25, 27). It should be noted that the kinetics of each affinity subpopulation differed markedly. The lowest affinity subpopulation, within the –Log aK ranges of 4.00–4.49, appeared early in the response (week 2), yet declined to undetected levels by week 6. The intermediate affinity subpopulations (–Log aK ranges of 4.50–5.99) appeared early, then declined over the response. The highest affinity subpopulations (–Log aK ranges of 6.00–6.99, or > 6.99) appeared next by week 6 or later and were maintained at a high percentage through week 18 (Figure 1C).

### TNP-Specific Production and Distribution (*ex vivo*) of ASCs

**Figure 2:**
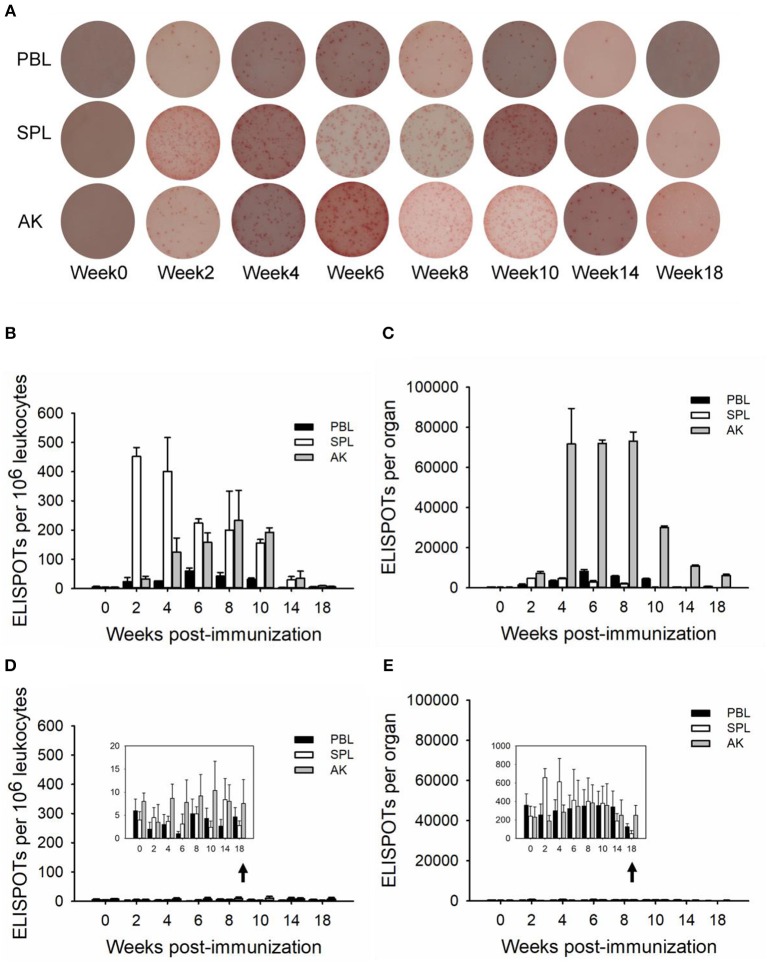
ELISPOTs of anti-TNP-specific antibody-secreting cells (ASCs) (*in vivo*) in immune tissues. The ELISPOTs of the *in vivo* ASC response within peripheral blood (PBL), spleen (SPL), and anterior kidney (AK) were shown from an individual fish immunized with TNP-KLH by 1 × 10^6^ leukocytes per well **(A)**. The average number of ASCs per 10^6^ leukocytes **(B,D)** and the total number of ASCs in each tissue **(C,E)** were detected at weeks 0, 2, 4, 6, 8, 10, 14, and 18 post-immunization (*n* = 5) with TNP-KLH **(B,C)** and PBS **(D,E)**. Data represent the mean ± SD of five individual fish at each time point and are representative of three independent experiments.

TNP-specific ASCs were monitored by ELISPOT from immune tissues over the 18-week immunization, allowing for visualization of the individual activated or responding cells (37). One million leukocytes were plated into each well, and the TNP-specific ASCs proportion occupied from one individual fish immunized with TNP-KLH is shown in Figure 2A. The spleen appeared to harbor the highest number of ASCs during the response as ASCs induced 10^6^ leukocytes, with a robust early response reaching the maximal value quickly at week 2 (Figure 2B). The anterior kidney was the most dominant ASC-inducing tissue during the response, maintaining a high level at weeks 4–8 (Figure 2C). The latter metric was important as the evaluation of the response should consider the kinetics of the ASCs at the level of the whole organ. At every time point, the anterior kidney was the primary contributor to the total number of *in vivo* ASCs, with a mean of 87.40% of the entire ASC response over the entire period. The mean blood contribution was ~7.81%, while the spleen only contributed 4.79%. The control group (immunized with PBS) did not exhibit any significant change over the process of the immunization (Figures 2D,E).

### Kinetics of Plasma Cells and LLPCs in Immune Tissues

**Figure 3:**
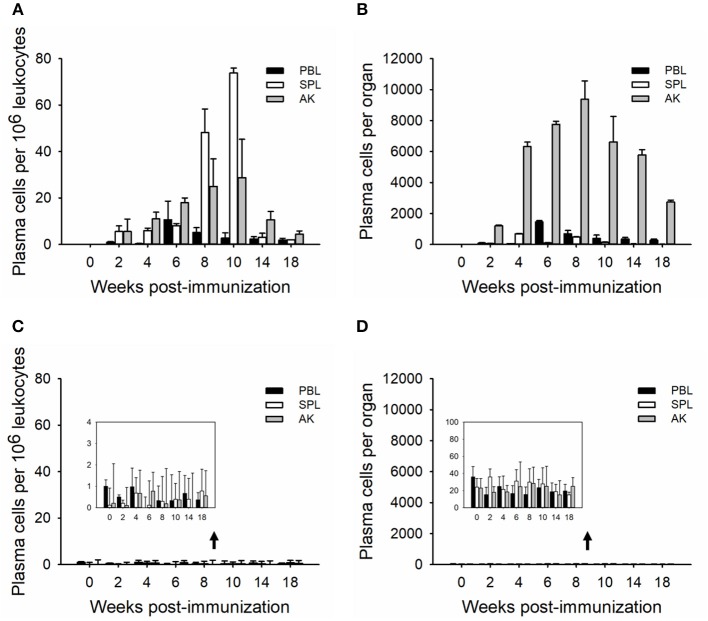
Kinetics of TNP-specific plasma (HU-insensitive, *in vitro* D7). The average number per 10^6^ leukocytes **(A,C)** and the total number in each tissue **(B,D)** detected in peripheral blood, spleen, and anterior kidney (*n* = 5) immunized with TNP-KLH **(A,B)** and PBS **(C,D)**. Data represent the mean ± SD of five individual fish at each time point and are representative of three independent experiments.

**Figure 4:**
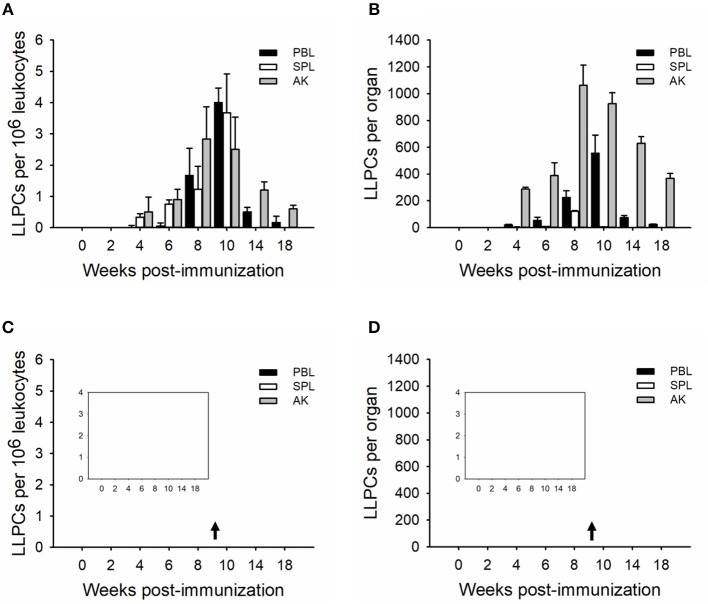
Kinetics of TNP-specific LLPCs (HU-insensitive, *in vitro* D15). The average number per 10^6^ leukocytes **(A,C)** and the total number in each tissue **(B,D)** detected in peripheral blood, spleen, and anterior kidney (*n* = 5) immunized with TNP-KLH **(A,B)** and PBS **(C,D)**. Data represent the mean ± SD of five individual fish at each time point time point and are representative of three independent experiments.

In order to determine the relative plasma cell response occurring in these immune tissues, leukocytes were cultured *in vitro* for seven (whole plasma cells) and 15 (LLPCs) days without antigen but in the presence of HU (Figures 3, 4). The technique with HU treatment permitted enumeration of non-replicating, antigen-insensitive plasma cells from other replicable cells. As shown in 10^6^ leukocytes, the plasma cell numbers responded much slower than the whole ASCs, with a small amount of TNP-specific plasma cells at week 2, which then reached the maximal in immune tissues at weeks 8–10 (Figures 3A,B). The control group did not exhibit any significant change over the process of the immunization (Figures 3C,D). Furthermore, the LLPCs appeared later than other subpopulations of ASCs (plasmablasts and SLPCs), with a generation beginning at week 4, and attained the peak as the plasma cells at weeks 8–10 (Figures 4A,B). The control group did not exhibit any significant change over the process of the immunization (Figures 4C,D). As for the whole organ, similar to the ASC response, the anterior kidney was the predominant contributor to the total number of plasma cells. In addition, the response of the anterior kidney plasma cells and LLPCs mirrored the antibody titers (Figure 1A), as well as the ASC responses (Figure 2C).

### LLPCs Secrete High-Affinity Antibodies

**Figure 5:**
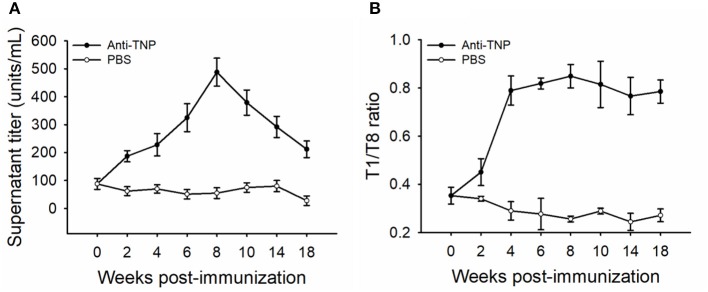
Kinetics of TNP-specific antibody titers and affinities secreted by LLPCs. The sample of detection was from the supernatant of HU-resistant plasma cells cultured *in vitro* for 15 days. The antibody titer **(A)** and affinity **(B)** shown are the average of five fish (*n* = 5). The PBS-immunized group was used as the control. Error bars represent standard deviation of the mean.

In order to determine the affinity of secreted antibodies by LLPCs, the supernatants of leukocytes from the AK cultured *in vitro* with HU addition for 15 days were collected, and TNP-specific titers and affinities were monitored. The dynamics of antibody titers in supernatants mirrored that in the sera, increasing rapidly at the early response and peaking at week 8 (~500 units/mL), then decreasing slowly during the late response (Figure 5A). The antibody affinities in the supernatants were examined by the T1/T8 affinity ELISA, with the analysis of the ratio of TNP_1_-BSA to TNP_8_-BSA values (28, 35). In accordance with the kinetics of antibody affinity in the sera, supernatant antibody affinity increased rapidly at week 2 and was optimal at weeks 6–8 and was then maintained at a steady high level (Figure 5B). With the HU presence for 15 days, the high-affinity antibodies in the supernatant were produced by LLPCs, which demonstrated that the LLPCs secreted the high-affinity antibodies late in the response.

### LLPCs Possess a High Antibody-Secreting Capacity

**Figure 6:**
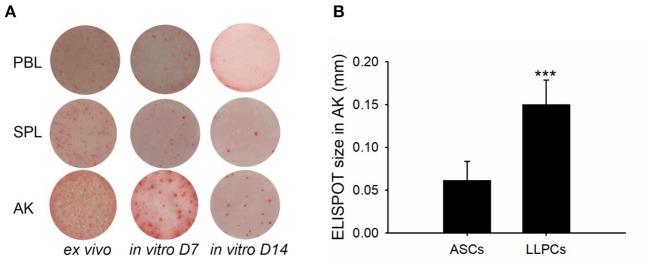
LLPCs (*in vitro* D15) have a strong capability to secrete antibodies compared to that of other ASCs *(ex vivo* and *in vitro* D7). ELISPOTs of TNP-specific ASCs from different immune tissues of vaccinated channel catfish 8 weeks post-immunization (2 × 10^6^ leukocytes per well) **(A)**. The average ELISPOT size of AK LLPCs relative to ASCs (*ex vivo*). Statistical difference was evaluated by *t*-test, and ***p < 0.001 **(B)**.

Leukocytes (blood, spleen, and anterior kidney) were cultured for 15 days in the absence of antigen but in the presence of HU, in order to determine the specific ASC antibody-secreting capacity. An analysis of the spot morphology indicated striking differences among the ASCs from various tissues (Figure 6A). The average spot size of AK LLPCs was significantly larger than ASCs (*ex vivo*) (Figure 6B). These data indicated that the LLPCs (non-replicating and antigen-insensitive) have a strong capability to secrete antibodies.

## Discussion

In this study, the existence of LLPCs was demonstrated in channel catfish, similar to mammals and rainbow trout (17), which survived *in vitro* for 2 weeks and continued to secrete antibodies for months post-immunization (16, 19, 24). The LLPCs began to appear at the early stage of immunization (4 weeks p.i.), resided mainly in the anterior kidney (bone marrow-like tissue) late in the immune response, and maintained the serum antibody response in channel catfish. More interestingly, as mammals, the antibodies secreted by LLPCs possessed high affinities in catfish. These observations might reveal that the LLPCs were selected by an affinity-driven mechanism within the survival niches of the anterior kidney to provide the long-term maintenance of high-affinity antibody titers in teleost fish.

The kinetics of serum antibody titers and antibody affinity in channel catfish were similar to the findings in rainbow trout (25, 27). Serum titers of TNP-specific antibodies reached the maximal level at week 6 post-immunization with a logarithmic increase and then began to decline (Figure 1A). This was similar to the findings in rainbow trout immunized with TNP-KLH (27) and channel catfish infected by *Streptococcus iniae* (38). The phenomenon of antibody affinity maturation occurred in channel catfish was detected by the T1/T8 affinity ELISA (Figure 1B), in accordance with the previous report that used an affinity partitioning ELISA (28). The binding ratio of different valency antigens to represent antibody maturation was first developed to measure the relative affinity of BCRs via a flow cytometry analysis (35). The theory that higher valence conjugates the binding of lower-affinity antibodies, but lower valence conjugates the binding of higher-affinity antibodies (35), had been used to separate different affinity IgM antibodies in rainbow trout and channel catfish (28, 39). The finding that lower affinity subpopulations disappeared while higher affinity subpopulations appeared later and maintained through week 18 confirmed the occurrence of affinity maturation in catfish (Figure 1C), indicating that the T1/T8 affinity ELISA was a solid and trustworthy assay.

Previous studies on channel catfish (40, 41) and rainbow trout (42, 43) have confirmed that leukocytes of specialized teleost immune tissues are fully capable of being induced by antigen *in vitro*. However, the actual *in situ* contribution of each of these tissues to the systemic antibody response can only be ascertained by monitoring the ASCs response *ex vivo*. Immunized with TNP-KLH, leukocytes from different immune tissues, including peripheral blood, spleen, and anterior kidney, were studied *ex vivo*, which performed differently in different tissues and analysis perspectives (Figure 2). The number of anti-TNP specific ASCs per 10^6^ leukocytes indicated that the primary tissues for the initial induction of the ASC response could be the spleen as it produced the greatest number of ASCs first (2 weeks p.i.) (Figures 2A,B). One corroborative observation is the temporary splenomegaly in response to antigenic challenge in juvenile turbot (44). To accurately gauge the contributions of different tissues, the total ASC response from each tissue was determined (19) in addition to determining the number of ASCs per 10^6^ leukocytes in this study, as it has been commonly done (38, 45, 46). Each tissue varied considerably according to the total number of leukocytes it possessed. The ASCs in the whole spleen, unlike in 10^6^ leukocytes, harbored a small part of ASC over the immunization period as compared to the blood and anterior kidney (Figure 2C). However, within 1–2 months (weeks 4–8 p.i.), a sizeable and persistent presence of ASCs within the anterior kidney occurred. The number of TNP-specific ASCs in whole peripheral blood peaked at week 6, which seemed to be in parallel to the anterior kidney and coincident with the antibody titer in the serum of TNP-specific IgM, indicating that the blood and anterior kidney were the main contributors to the serum antibody response at this time, as in rainbow trout (47).

B cell developmental pathways in teleost fishes are poorly understood in the absence of serological reagents; however, the use of transcription factors that are differentially expressed during B cell development helps to dissect teleost B cell development, which defined B cell subsets during terminal B cell differentiation as resting B cells, activated B cells, plasmablasts, and plasma cells (48–51). Based on the longevity, plasma cells were classified as the SLPCs and LLPCs, and HU was used to distinguish plasmablast (HU-sensitive) and plasma cell (HU-insensitive) activities (17, 47). As in trout, leukocytes were cultured with HU *in vitro* for 7 and 15 days to ascertain the number of plasma cells vs. LLPCs (17, 19, 52, 53), respectively (Figures 3, 4). Generally, the number of plasma cells in the spleen and anterior kidney was higher than in blood per 10^6^ leukocytes over the immunization (Figure 3A). Recent studies have demonstrated that the spleen is primarily composed of the early differentiative stage, the plasmablasts (48), which then differentiate into PCs. In teleosts, the anterior kidney, which serves as the bone marrow of mammals, contains different stages of differentiated B cells, which, aside from the hematopoietic tissue of the fish, also serves as a reservoir for plasma cells (17, 19, 52, 53). Peripheral blood contained most of the resting B cells and served a greater critical role in delivering antigen and B cells to other immune tissues (47, 48), indicating that the plasma cells in the blood may migrate from other immune tissues, such as the spleen and anterior kidney. Total plasma cells in immune tissue after immunization revealed that the anterior kidney was the contributor providing persistent antibody response (Figure 3B). Different from plasma cells, LLPCs appeared at week 4 post-immunization (Figure 4) and mostly resided in the anterior kidney, with a small part occurring in the blood and spleen. The migration of LLPCs here implied that, though a small part of LLPCs existed in the blood and spleen, the anterior kidney served as a reservoir for LLPCs as in rainbow trout during the late response (17, 19). Thus, the two functions, hematopoiesis and LLPC niche, coincidentally occurred within the anterior kidney in teleosts, which is similar to what occurs in the mammalian bone marrow (54, 55). In mammals, at the late stage of the response, a high-affinity plasma cell population that accumulated in the bone marrow was confirmed to be long-lived and responsible for the long-term systemic production of high-affinity antibodies required for protective immunity (11). In this study, the LLPCs in catfish were also proven to secrete high-affinity antibodies (Figure 5) as in mammals, with the strong antibody-producing capability (Figure 6).

**Figure 7:**
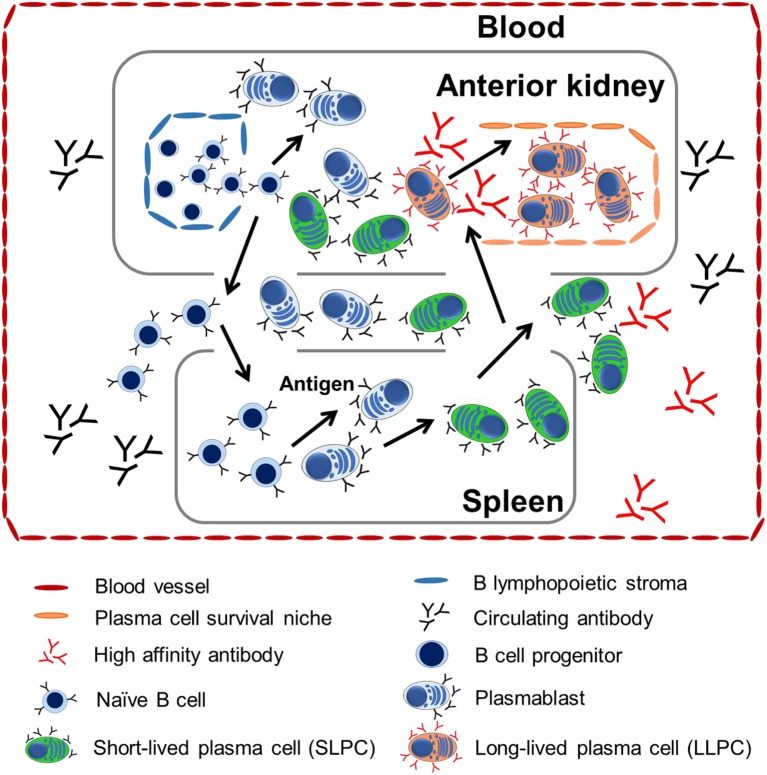
A proposed model of tissue distribution and migration of LLPCs from teleosts. Anterior kidney is the bone marrow-like tissue posing the major site of B lymphocyte development where B cell progenitors and naive B cells arise. They migrate to peripheral tissue via the blood. When these B cells encounter antigen, they mature into plasmablasts or PCs in either the anterior kidney or spleen. Plasmablasts or PCs differentiate within the spleen and anterior kidney, but home to the anterior kidney, wherein PCs from all tissues compete for long-term maintenance within the survival niches in the anterior kidney.

Our data revealed that there was a positive linear correlation between the survival environment and cell property with the maintenance of long-term serum antibody titer in channel catfish. The LLPCs may contribute to one of the mechanisms maintaining teleost humoral immunological memory as in mammals (3, 56). A model of the distribution and migration of LLPCs was proposed from the data presented and from previously published work on ASC development in rainbow trout (16, 17, 19, 47) (Figure 7). As in mammals, the naive B cells of teleosts first mature within the hematopoietic-like tissues (anterior kidney) and B lymphopoietic stroma from their pro-B and pre-B progenitors. Mature naive B cells migrate via the peripheral blood into other immune tissues, such as the spleen. They differentiate into plasmablasts when they encounter the antigen, which accounts for the initial humoral immune response observed. After proliferation, the plasmablasts may differentiate into PCs. Upon receiving specialized cues (57, 58), PCs may migrate and then home and situate themselves within the anterior kidney survival niches as LLPCs. LLPCs secrete high-affinity antibodies into peripheral circulation to maintain the serum antibody titer at a steady level, which may indicate that the challenge to become LLPCs in teleosts is driven by affinity selection (29, 39) as reported in mammals.

This work demonstrates that LLPCs secrete high-affinity antibodies to sustain the titer and affinity in the late immunization. LLPCs are not uniformly distributed in the lymphoid organs of channel catfish but are specifically located in the anterior kidney (bone marrow-like tissue) with a large proportion during the immunization. The discovery of the LLPCs and high-affinity antibodies secreted from LLPCs in channel catfish has many implications for the design and delivery of vaccines in teleosts. Successful vaccination may require long-term maintenance of high-affinity antibodies produced by the plasma cells within the anterior kidney. Given that long-term protection to certain pathogens may ultimately depend on the maintenance of LLPCs, it is essential that the mechanisms of LLPC generation, development, and maintenance in the anterior kidney must be delineated. Moreover, the finding of LLPCs secreting high-affinity antibodies may shed light on the evolutionary understanding of the affinity-dependent mechanism of the generation and development of LLPCs.

## Data Availability Statement

The datasets analyzed in this manuscript are not publicly available. Requests to access the datasets should be directed to jmye@m.scnu.edu.cn.

## Ethics Statement

The animal study was reviewed and approved by University Animal Care and Use Committee of the South China Normal University.

## Author Contributions

LW performed the majority of the experimental work, with the assistance of SF in feeding the fish and performing the ELISPOT experiment. XY performed data analysis and graphing. ZG and AW reviewed and polished the manuscript. JY and LW designed the experiments and wrote the main body of the paper.

### Conflict of Interest

The authors declare that the research was conducted in the absence of any commercial or financial relationships that could be construed as a potential conflict of interest.
